# A Novel *KCNJ2* Mutation Identified in an Autistic Proband Affects the Single Channel Properties of Kir2.1

**DOI:** 10.3389/fncel.2018.00076

**Published:** 2018-03-20

**Authors:** Anna Binda, Ilaria Rivolta, Chiara Villa, Elisa Chisci, Massimiliano Beghi, Cesare M. Cornaggia, Roberto Giovannoni, Romina Combi

**Affiliations:** ^1^School of Medicine and Surgery, University of Milano-Bicocca, Monza, Italy; ^2^Department of Mental Health, AUSL Romagna, Ravenna, Italy

**Keywords:** autism spectrum disorders, *KCNJ2*, potassium channel, mutation, patch clamp, single channel

## Abstract

Inwardly rectifying potassium channels (Kir) have been historically associated to several cardiovascular disorders. In particular, loss-of-function mutations in the Kir2.1 channel have been reported in cases affected by Andersen-Tawil syndrome while gain-of-function mutations in the same channel cause the short QT3 syndrome. Recently, a missense mutation in Kir2.1, as well as mutations in the Kir4.1, were reported to be involved in autism spectrum disorders (ASDs) suggesting a role of potassium channels in these diseases and introducing the idea of the existence of K^+^ channel ASDs. Here, we report the identification in an Italian affected family of a novel missense mutation (p.Phe58Ser) in the *KCNJ2* gene detected in heterozygosity in a proband affected by autism and borderline for short QT syndrome type 3. The mutation is located in the N-terminal region of the gene coding for the Kir2.1 channel and in particular in a very conserved domain. *In vitro* assays demonstrated that this mutation results in an increase of the channel conductance and in its open probability. This gain-of-function of the protein is consistent with the autistic phenotype, which is normally associated to an altered neuronal excitability.

## Introduction

Inwardly-rectifying potassium channels (Kir) are widely expressed in several excitable and non-excitable tissues playing a key role in the maintenance of the resting membrane potential and consequently in the regulation of cell excitability. Approximately 15 Kir clones were identified and grouped in seven different families based on sequence similarity and functional properties: Kir1.x—Kir7.x. Kir2.x family includes five isoforms sharing the biophysical characteristic of strong inward rectification due to a highly voltage-dependent block of the channel pore by intracellular polyamines and Mg^2+^ (Lopatin et al., 1994; Fakler et al., 1995). In particular, Kir2.1 (IRK1/*KCNJ2*) is the major isoform underlying the inward-rectifier current IK1 in the human ventricular muscle (Melnyk et al., 2002). High levels of this isoform were also reported in the brain (mainly hippocampus, caudate, putamen and nucleus accumbens; Karschin et al., 1996) and in the skeletal muscle.

Mutations in *KCNJ2* leading to loss-of-function of Kir2.1 have been linked with Andersen-Tawil syndrome, a cardiovascular disease characterized by QT prolongation, predisposition to cardiac tachyarrhythmias (Donaldson et al., 2004) as well as skeletal abnormalities, mood disorders and seizures (Guglielmi et al., 2015). On the other hand, Kir2.1 gain-of-function mutations cause the type-3 variant of the short QT syndrome (SQT) which results in QT shortening and increased risk of sudden cardiac death (Priori et al., 2005).

Recently, a role of Kir channels was reported in autism spectrum disorders (ASDs) due to the identification of two mutations (p.Arg18Gln and p.Val84Met) in the *hKCNJ10* gene coding for the Kir4.1 subunit as well as one mutation in the *KCNJ2* gene (p.Lys346Thr) coding the Kir2.1 subunit (Sicca et al., 2011). All of these mutations resulted in a gain-of-function of the relevant Kir channel (Guglielmi et al., 2015; Sicca et al., 2016). ASDs are heterogeneous disorders characterized by a neurodevelopment impairment with complex etiology: several environmental risk factors have been reported and a strong genetic basis is accepted with an estimated heritability upwards of 90% (Bailey et al., 1995). Nonetheless, autism specific genetic etiology remains largely unknown.

Here, we report the identification and the functional characterization of a novel missense mutation (*KCNJ2*- p.Phe58Ser) located in the coding region of the *KCNJ2* gene in an ASD patient showing a borderline shortening of the QT interval at the electrocardiogram (ECG).

## Materials and Methods

### Genetic Analysis

An Italian family showing three cases of ASDs and several cases of different cardiovascular diseases composes the sample.

In accordance with the Declaration of Helsinki, all participants in the study signed an informed written consent in accordance with the study protocols approved by the University of Milano-Bicocca ethical committee.

For each participant genomic DNA was obtained using the Wizard genomic DNA purification kit (Promega, Madison, WI, USA) from venous blood.

Comprehensive mutational analyses of *KCNJ2* and *KCNJ10* were performed using polymerase chain reactions (PCRs) and DNA sequencing. PCRs were performed under standard conditions directly on genomic DNA using the GoTaq Master Mix (Promega). Flanking primers (Sigma-Aldrich, St. Louis, MO, USA) designed with the Oligo 6.0 software were used to amplify *KCNJ2* and *KCNJ10* coding sequences. Sequencing reactions were performed on both strands using the BigDye Terminator Cycle Sequencing kit v1.1 and an automated ABI-3130 DNA sequencer (Applied Biosystems, Foster City, CA, USA). Mutation detection was performed using ChromasPro v1.34 (Technelysium Ltd.) software. Sequences were compared with the RefSeq sequences NM_000891 (*KCNJ2*) and NM_002241.4 (*KCNJ10*).

### Plasmids and Mutagenesis

A pCMS-EGFP vector containing the wild-type (WT) *KCNJ2* cDNA was kindly provided by Prof. Minoru Horie (Shiga University of Medical Science, Japan; Haruna et al., 2007). The c.173T>C (p.Phe58Ser) mutation was introduced by site-directed mutagenesis using the Quick Change II XL kit (Stratagene, La Jolla, CA, USA). The cDNA was completely resequenced after mutagenesis. WT or mutant cDNA was sub-cloned into a pcDNA3.1-NT-GFP-TOPO vector (Invitrogen), so that the GFP protein was fused at the N-terminus of the Kir2.1 channel. Plasmids were purified using QIAGEN Plasmid Maxiprep kit (QIAGEN, Hilden, Germany) following the suggested protocol. All constructs were verified by sequence analysis.

### Cell Culture and Transfection

tsA201 cell line (purchased from Sigma-Aldrich) was used for heterologous expression. Cells were cultured in a controlled environment (5% CO_2_, 37°C) and maintained in Dulbecco’s Modified Eagle Medium (DMEM; Euroclone, Italy) supplemented with fetal bovine serum (10%), L-Glutamine (2 mM), penicillin G (100 U/mL) and streptomycin sulfate (100 μg/mL). Cells were transiently transfected with 1 μg of pcDNA3.1-NT-GFP-TOPO-h*KCNJ2*-WT or pcDNA3.1-NT-GFP-TOPO-h*KCNJ2*-p.Phe58Ser, coding for WT or mutant Kir2.1 channel, using jetPRIME reagent (Polyplus Transfection, Euroclone) according to the manufacturer protocol. All experiments were carried out 48 h after transfection and untransfected tsA201 cells were used as a negative control.

### Immunoblotting Analyses

Briefly, tsA201 cells were detached by scraping the plate surface collected for immunoblot analyses.

Total protein extracts were obtained by lysing the cells with 1× RIPA lysis buffer (50 mM Tris-HCl pH 7.4, 150 mM NaCl, 1% Triton X-100, 0.1% SDS) added with 1 mM DTT, 1 mM EDTA and EGTA, and 1.5% Protease Inhibitor Cocktail and Phosphatase Inhibitor Cocktail. Protein extracts were quantified by Bradford assay (Sigma-Aldrich).

Ten microgram of total protein extracts were loaded on NuPAGE Bis-Tris pre-casted mini gels (Life Technologies), following manufacturer instructions. Blotting onto nitrocellulose membrane (Life Technologies) was performed using iBlot System 2 (Life Technologies). Nitrocellulose membranes were blocked with 5% milk solution prepared in PBS-Tween 0.1% and then incubated with anti-Kir2.1 (mouse monoclonal [S21–32], 1:1000; Abcam [ab85492]) and anti-β-actin (mouse monoclonal [AC-15], 1:5000, Sigma Aldrich) antibodies for 1 h at room temperature. Membranes were washed three times in PBS-Tween 0.1% and then incubated with ECL anti-mouse IgG HRP linked secondary antibodies (1:5000, GE Healthcare). After three washes in PBS-Tween 0.1%, Liteblot^®^ Extend Long Lasting Chemiluminescent Substrate (Euroclone) was added to the membranes and chemiluminescent signal was digitally acquired by GBox (Syngene). Densitometry analyses of Western blot bands were performed by using “Gel analyzer” function of ImageJ software (Schneider et al., 2012).

### Immunocytochemistry and Imaging

For localization studies, tsA201 cells were plated in 35 mm glass-bottom dishes, transiently transfected and fixed in methanol for 6 min at −20°C after which they were washed three times with high salt buffer (10 min each) and incubated overnight at 4°C with Anti-Kir2.1 (mouse monoclonal, [S21–32], 1:100, Abcam [ab85492]) and anti-ZO1 (rabbit polyclonal, 1:50; Invitrogen) in GDB. The secondary antibody was conjugated with Alexa fluorophores (goat anti-mouse Alexa Fluor 488, goat anti-rabbit Alexa Fluor 568; Invitrogen) diluted in GDB, at room temperature for 1 h. Wheat Germ Agglutinin (WGA) was incubated for 1 h at RT (1:200, Thermo Fisher). One micromolar of 4’,6-diamidino-2-phenylindole (DAPI) in PBS stained cell nuclei (5 min). Confocal laser scanning microscopy was used to study the proteins cellular distribution. Images were acquired by an LSM710 inverted confocal microscope equipped with a Plan-Neofluar 63× 1.4 oil objective (Carl Zeiss, Germany). The intensity of the green and the red signals (λem = 488 nm and 610 nm, respectively) at the plasma membrane were evaluated using ImageJ software: after selecting a ROI (Region Of Interest) on the acquired images, the fluorescence was expressed as arbitrary units (a.u.). The a.u. values associated with Kir2.1 protein were normalized to the ones associated with the constitutive proteins (or glycoproteins in case of WGA) present on the plasma membrane. λem = 460 was used to detect the nuclei.

### Patch-Clamp Recordings and Analysis

Patch clamp experiments were performed at room temperature using pipettes pulled to a resistance of 2–5 MΩ for whole-cell recordings and 7–8 MΩ for single-channel ones (Model P-97, Sutter Instruments, Novato, CA, USA). Pipettes were filled with an intracellular solution containing (in mM): KAsp 132, KCl 15, MgCl_2_ 1, HEPES 5 (pH 7.3 with KOH). For whole-cell measurements tsA201 cells medium was replaced with an extracellular solution containing (in mM): NaCl 135, KCl 4.8, CaCl_2_ 1.8, MgCl_2_ 1, HEPES 5, glucose 10 (pH 7.4 with NaOH). BaCl_2_ 1 mM was added to the bath solution to selectively block the inward rectifying current. For single channel currents, recorded using the cell-attached configuration of the patch-clamp technique, the extracellular solution contained (in mM): KCl 140, MgCl_2_ 1, CaCl_2_ 1.5, HEPES 10, glucose 10 (in this case, high potassium was present in the pipette and in the bath solution in order to set the intracellular potential near 0 mV).

Acquisitions were made with a Multiclamp 700B amplifier (Axon Instruments, Molecular Device, Sunnyvale, CA, USA); whole cell currents were sampled at 10 kHz, low-pass filtered (1 KHz, Bessel 8-pole filter, −3 dB), digitized with a Digidata 1440A (Axon Instruments) and pClamp 10.3 software (Molecular Devices) was used for the analysis. Whole-cell IK1 data were plotted as barium-sensitive currents.

For the analysis of the single-channel properties, currents were filtered with a Butterworth filter (8-pole), −3 dB with a cutoff frequency of 0.5 KHz (Silberberg and Magleby, 1993). Openings and closures were identified by the “half-height” criterion. Channel conductance was obtained as the slope of the I-V relationship applying potential of −100, −120 and −140 mV (Qu et al., 2015). Open probability was calculated from the total open times divided by the total sweep duration.

### Statistical Analysis

Data are presented as mean ± SEM. Two-tailed Students *t*-test was used to compare means; *p* < 0.001 was considered statistically significant and indicated with *.

## Results

### Phenotypes

**Figure 1:**
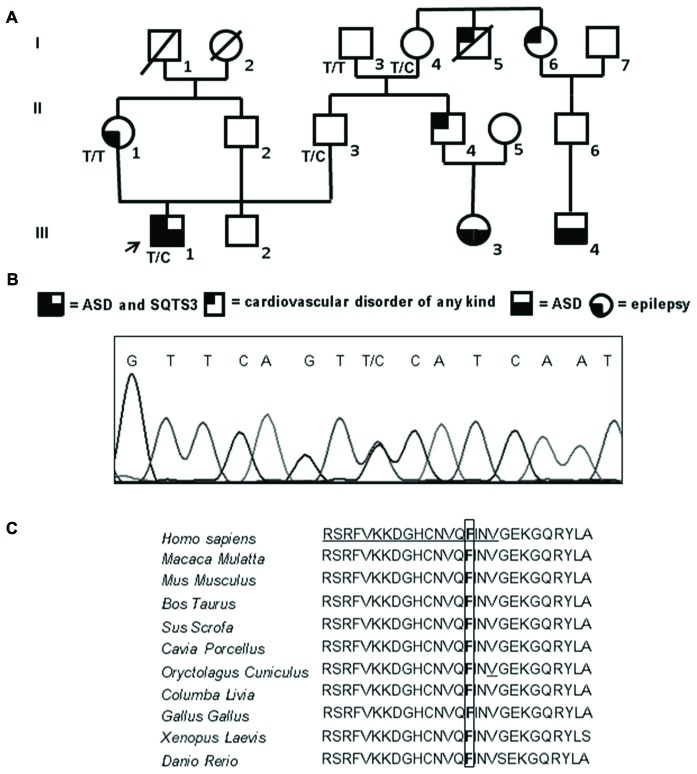
Familial genetic history, electropherogram of the proband and schematic representation of the ion channel. **(A)** Pedigree of the family in which the mutation has been identified showing the segregation of the c.173 T>C (p.Phe58Ser) mutation in collaborative relatives. The arrow indicates the proband. Genotypes for compliant individuals are shown. T/T: wild-type (WT) genotype; T/C: heterozygous genotype. Squares indicate males while circles indicate females. The legend of each kind of symbol filling (independently from the represented gender) is reported. **(B)** Electropherogram from the proband heterozygous for the transition c.173 T>C (RefSeq NM_000891) that corresponds to the missense mutation p.Phe58Ser. **(C)** Amino acid multiple alignment of Kir2.1 sequence displaying evolutionary conservation of Phenylalanine F residue across species. The mutant F at position 58 is boxed and the Golgi export signal is underlined in the human sequence.

The proband (individual III-1, Figure 1A) is now a 23 years old man with an ASD diagnosed during the attendance of infant school when he showed a language delay (he started to speak at the age of about three years) as well as a gestural automatism in the form of “flicker” (with the arms makes the gesture of flying repeatedly). Moreover, he was hyperactive and had severe relationship problems. Currently he finished an art school and worked as a storekeeper in a large supermarket. He’s still followed by psychiatrists and educators in an educational and job placement project for people with disabilities. For a long time he lived basically isolated and with few interpersonal relationships. The ECG examination (not shown) performed on the proband after the identification of the mutation reported here showed a borderline shortening of the QT interval (QTc of 320 ms and QRS of 120 ms).

The father (individual II-3, Figure 1A) is a healthy man of 54 years with only rare episodes of childhood asthma in his history; his schooling is up to high school without any evident problems concerning performance or interpersonal relationships. He is currently employed in a large company and has a life of relationship substantially normal. No detailed psychological tests were available for this subject.

Both paternal grandfather (individual I-3, Figure 1A) and grandmother (individual I-4, Figure 1A), 82 and 80 years old respectively, are alive; the grandfather was always in good health (it’s only reported the presence of gout in recent years); the grandmother, from the age of 45–50 years reported episodes of angina pectoris. Her brother (individual I-5, Figure 1A) died of a sudden cardiac arrest while sleeping at around 50 years old without any previous warning, while her sister (individual I-6, Figure 1A) was operated to heart last year and has a nephew (individual III-4, Figure 1A; son of the son) with an important and diagnosed autism spectrum disorder with severe disorganization of development. No detailed psychological tests were available for these individuals.

The brother (individual II-4, Figure 1A) of proband’s father, born in 1966, takes Cardioaspirin for not well defined heart problems. The daughter (individual III-3, Figure 1A) of this brother, who is now 7 years old, has been under scrutiny for an autism spectrum disorder: she has a language delay, gestural automatism in the form of “flicker” (the same automatism of the proband) and she has little relationships and communication skills.

The mother (individual II-1, Figure 1A) of the proband is a 51 years old woman with a history of idiopathic generalized epilepsy with age-related onset, generalized tonic-clonic seizure and photosensitivity; the epilepsy onset was reported at age 11 while at present she has rare attacks. The average schooling is superior and she is currently enrolled in a public company. Her father (individual I-1, Figure 1A) died at the age of 94 years old and her mother (individual I-2, Figure 1A) at 66 years old for cancer, not having presented particular diseases in life.

### Mutational Screening

Sequencing of the coding region, intron-exon boundaries of *KCNJ2* revealed that the proband is a heterozygote for a missense mutation (Figure 1B). Nucleotide numbering from here onward is according to cDNA position (RefSeq accession number NM_000891 starting from the first nucleotide of the ATG start codon).

The mutation consists of a T>C transition at cDNA position 173 (c.173T>C), which leads to a non-conservative Phe to Ser change at position 58 (p.Phe58Ser) in the N-terminal cytoplasmic domain of Kir2.1. This mutation was detected in the heterozygous state also in the proband’s father (II-3) who inherited the mutated allele from his mother (I-4), while it was absent in the epileptic proband’s mother (II-1; Figure 1A). Both the father and the grandmother are not affected by ASDs. Unfortunately, parents (II-4 and II-6) of the other affected children in the family (III-3 and III-4) were not collaborative, thus a cosegregation study between the mutation and ASDs was not feasible. The mutation was not previously reported in literature nor present in SNP databases. The amino acid change in Kir2.1 was predicted to be damaging (PolyPhen2, Mutation Taster) and affected a highly evolutionary conserved amino acid (Figure 1C). No mutations were detected in the coding region of *KCNJ10*.

### Expression Level and Localization of Wild Type and Mutated Channel

**Figure 2:**
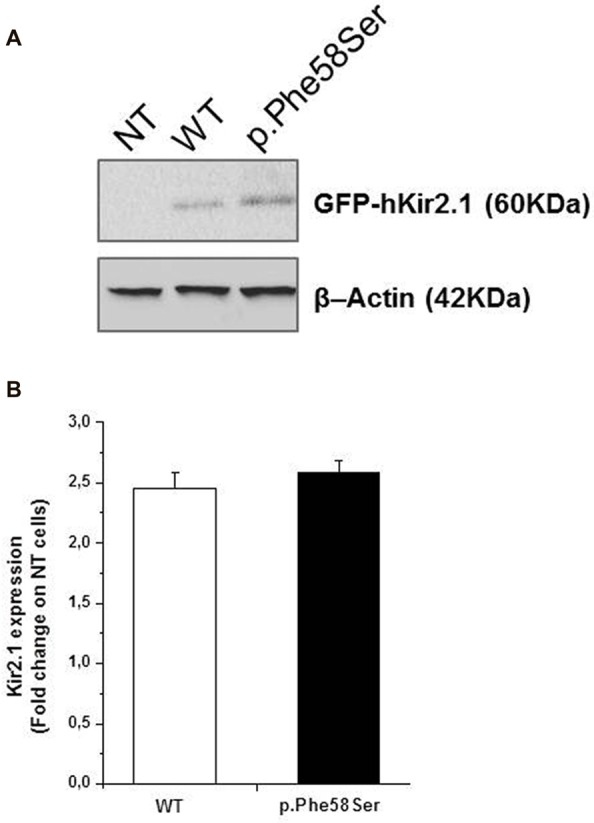
Expression level of WT and p.Phe58Ser channel. **(A)** Immunoblotting analysis on total protein extracts from untransfected tsA201 cells (NT), tsA201 cells transfected with pcDNA3.1-NT-GFP-TOPO-hKCNJ2-WT plasmid (WT) or with pcDNA3.1-NT-GFP-TOPO-hKCNJ2-p.Phe58Ser plasmid (p.Phe58Ser) is showed. β-Actin was used as endogenous controls of equal protein load. **(B)** Densitometry analysis of Kir2.1 expression levels in total protein extracts from tsA201 cell lines. Data are expressed as fold change ratio on untransfected cells (NT) where p.Phe58Ser and WT indicate the relative protein expression in cells transfected with the mutant or WT encoding plasmids, respectively, normalized to the β-Actin protein expression levels. Data shown are representative of five independent experiments.

In order to investigate whether p.Phe58Ser-mutated Kir2.1 recalls a previously reported mutation in *KCNJ2* gene related to ASD, which exhibited an increment in the ion channel protein levels (Ambrosini et al., 2014), total protein extracts were analyzed via western blotting by using specific anti-Kir2.1 antibody and the relative protein abundance was quantified by densitometry measurements. The analysis showed an approximately similar amount of the exogenous proteins independent of the presence of the mutation, being 2.45 ± 0.1 and 2.59 ± 0.1 the fold change of the Kir2.1 expression of the WT and the p.Phe58Ser respectively vs. the untransfected cells (Figure 2).

As Kir2.1 protein was fused with GFP, perfectly co-localized signals at the molecular weight of about 60 kDa were observed when nitrocellulose membranes were probed (and detected) with an anti-Kir2.1 (Chemioluminescent detection) and an anti-GFP antibody (fluorescent assay; see Supplementary Figure S1).

**Figure 3:**
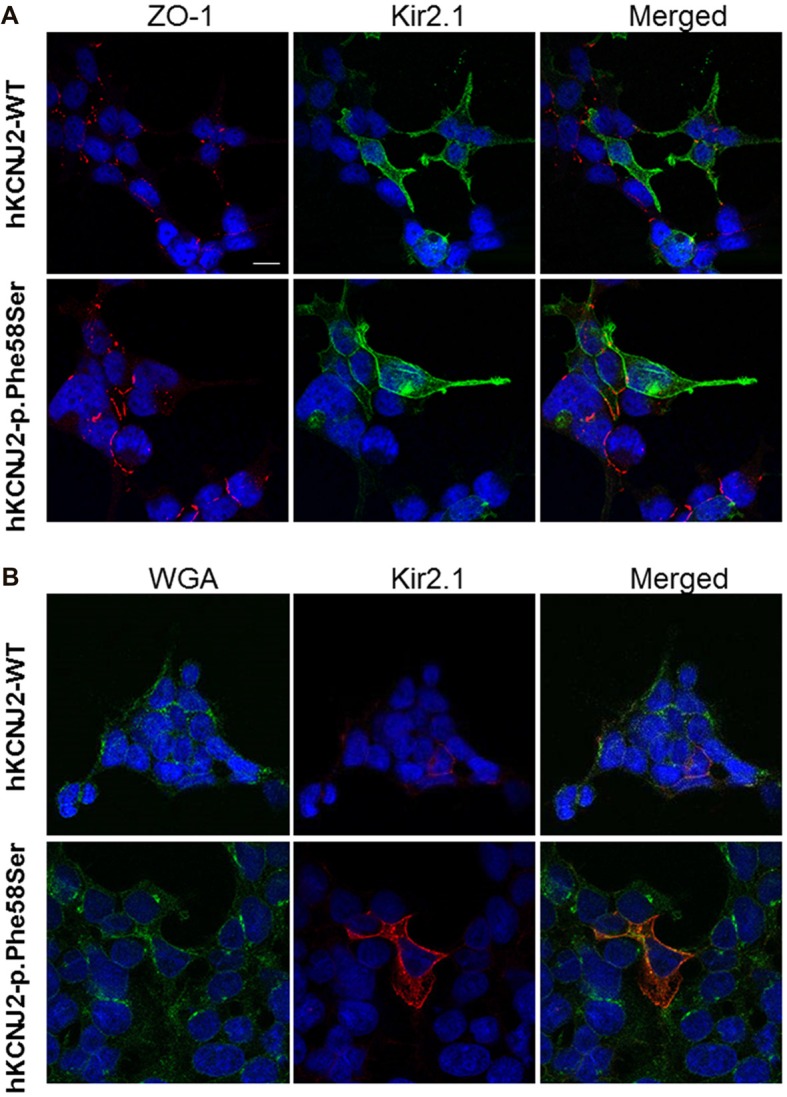
Immunofluorescence staining of tsA201 cells expressing either WT or p.Phe58Ser *KCNJ2*. In **(A)**, images in the left column represent zonula occludens 1 (ZO-1) labeled in red, in the central column Kir2.1 is stained in green. In **(B)** instead, images in the left column represent WGA membrane staining labeled in green and in the central column Kir2.1 stained in red. In both the panels, the right columns represent the merged images. Nuclei were stained in blue. The scale bar is 10 μm. Images shown are representative of one of three independent experiments.

A previous study demonstrated that the introduction of the GFP at the N-terminus of the *KCNJ2* cDNA did not affect its targeting (Eckhardt et al., 2007). Based on the similar amount of WT or p.Phe58Ser GFP/KCNJ2 protein found in transfected cells, we then investigated for a possible difference in sub-cellular localization by analyzing cytosolic and membrane fractions of transfected cells. We found a trend, although not significant, of higher abundancy in membrane compartment of p.Phe58Ser *GFP/KCNJ2* expressing cells as compared to WT *GFP/KCNJ2* expressing cells (see Supplementary Figure S2). In order to gain a single cell resolution analysis on the possible higher abundance of channels on the surface membrane, we performed co-immunofluorescence experiments using zonula occludens 1 (ZO-1), a protein associated to tight junction and expressed by tsA201 cells, as membrane marker to standardize the ion channel quantification. Confocal imaging revealed for both WT and mutant channels a clear membrane localization, and a minor intracellular labeling (Figure 3A). The mutated channel was found to have a slight but significant increase in plasma-membrane localization, as suggested by the fluorescence quantification: 0.77 ± 0.21 for WT and 0.88 ± 0.25 for p.Phe58Ser (*n* = 351 and 342 images, respectively, from three different experiments, *p* < 0.001). Similar results were obtained when WGA, was used as plasma membrane marker instead of ZO-1 (Figure 3B). In this case the quantification yielded 2.01 ± 0.5 and 2.20 ± 0.3 for the WT and p.Phe58Ser Kir2.1 channel, respectively (*n* = 25 images). These findings suggest that the mutated channel has an increased localization to the membrane as compared to the WT protein. However, this slight difference is not quantitatively comparable with the expectation based on previous data regarding other mutations (Eckhardt et al., 2007) and could not account for the observed pathological phenotype.

### Cellular Electrophysiology

In order to study closely the behavior of the mutated protein as compared to the WT one, we therefore moved to a functional approach. As the constructs used for the cells transfection in all the experiments (western blot, immunocytochemical and electrophysiological) tagged the *KCNJ2* gene with the GFP fused at the N-terminus of the protein, we firstly verified and confirmed that this fusion did not alter the whole-cell current properties (see Supplementary Figure S3).

**Figure 4:**
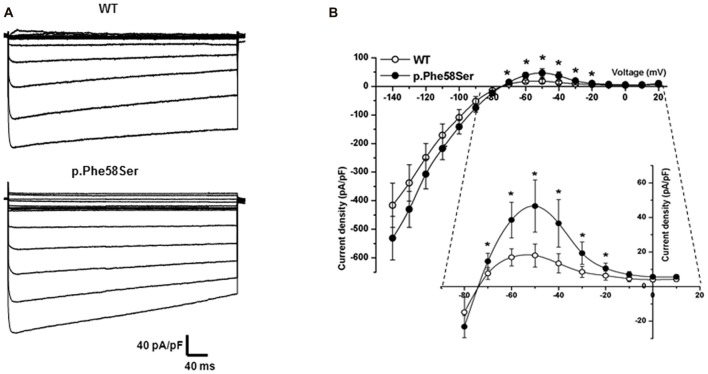
Functional characterization of WT and mutant Kir2.1. **(A)** Typical families of IK1 barium-sensitive current traces recorded in whole-cell from tsA201 cells transfected with WT (upper) and p.Phe58Ser-mutated (lower) channels. **(B)** Current-Voltage relation showing the averaged current densities (*n* = 27 for WT, empty circles and *n* = 20 for the mutant, filled circles).

Whole-cell IK1 currents were acquired through a steps protocol of the duration of 400 ms with a holding potential of −60 mV and steps amplitude from −140 mV to +20 mV. In Figure 4 barium-sensitive current traces from tsA201 cells expressing WT or mutant *KCNJ2* (Figure 4A, upper and lower families of currents, respectively) are shown. Kir2.1-p.Phe58Ser channels generated a significant higher current density compared to the WT ones (Figure 4B), in particular in the range of the inward rectification voltages, between −70 mV and −20 mV, as can be better appreciated from the inset in the figure. At −50 mV, when the outer current is at its maximum, we measured 46.6 ± 15.1 pA/pF in cell expressing the mutant and 18.0 ± 6.7 pA/pF in those expressing the WT cDNA (*p* < 0.001). No significant differences were found between the current densities measured at the peak or at the end of the protocol pulse for the WT or mutant channels. Consistent with this result, the resting membrane potential (V_rest_) of the cells transfected with the Kir2.1-p.Phe58Ser was on average hyperpolarized compared with the cells transfected with the WT channel, being −68.06 ± 1.2 mV and −62.22 ± 9.7 mV, respectively (*p* < 0.05; *n* = 34 for both the conditions from three independent experiments. The V_rest_ of untransfected cells was −9.3 ± 2.8, *n* = 19).

**Figure 5:**
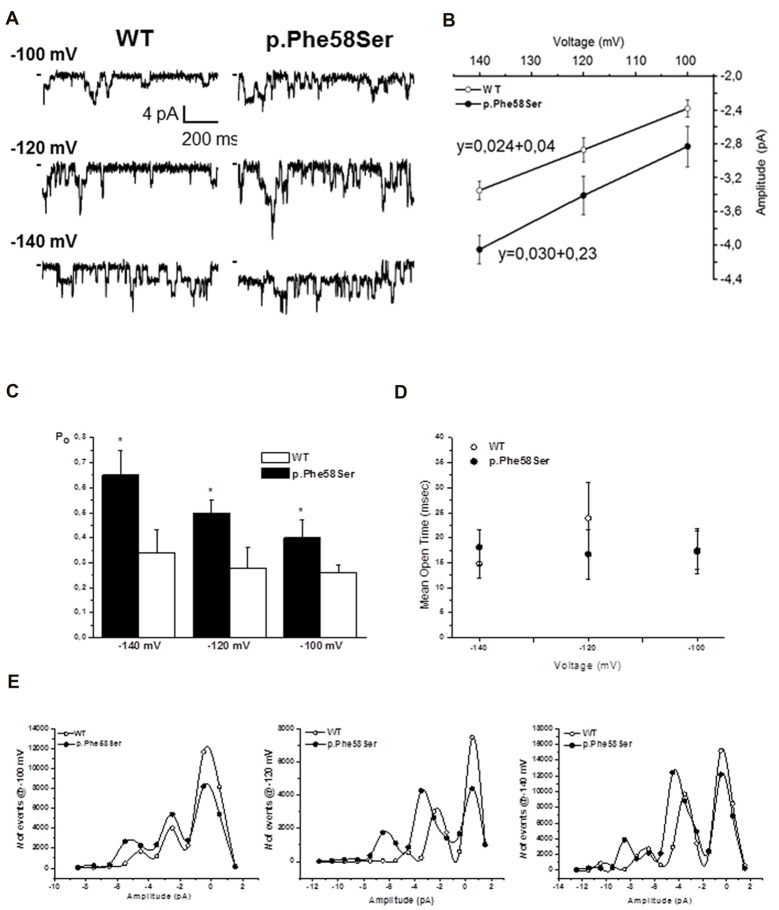
Single channel properties. **(A)** Representative single-channel current traces obtained at different voltages from a given patch containing active Kir2.1 WT or p.Phe58Ser channels. **(B)** Current-voltage relation obtained plotting the unitary single-channel current amplitude against the voltage (*n* = 3–5 patches for each experimental condition). Solid lines represented fits of the linear regression equation, that is shown, with the slope being the single channel conductance. **(C)** Open probability obtained at different voltages for the WT (white column) and p.Phe58Ser (black column) Kir2.1. **(D)** Mean open time measured for the WT (open circles) and p.Phe58Ser (filled circles) channels. **(E)** Distribution of the events at different voltages vs. the amplitude of the current showing a higher number of opening events for the mutated channel (filled circle) compared to the WT (empty circle). As the peak of the opening events is shifted towards larger current for the p.Phe58Ser, these data also recall the mutation-induced larger conductance.

The western blot and immunofluorescence imaging could not justify the larger current in terms of mutation-induced higher protein abundance at the plasma membrane level. Therefore, in order to better investigate the gain-of-function effect of the mutated channel, we performed recording in patch-clamp cell-attached configuration. Considering the overexpression of the Kir2.1 proteins, it was difficult to find patches with one single channel, more often we saw two or three channels in a given patch. Current traces in Figure 5A are representative of patches with at least three channels. On average, the single-channel current amplitude recorded in p.Phe58Ser channel was significantly larger than that in WT: (−3.4 ± 0.2 pA and −2.8 ± 0.1 pA, respectively at a potential of −120 mV, *n* = 3 patches, *p* < 0.001). Measurements were conducted also at −100 and −140 mV and unitary conductance of 24 pS for the WT channel and 30 pS for the mutant one was obtained from the slope of the current-voltage relationship (Figure 5B). Results also showed that the mutation increased the mean open probability but not the mean open time of the channel at all the three voltage tested (Figures 5C,D). In particular, the open probability (Po) calculated at −120 mV was 0.28 ± 0.08 and 0.5 ± 0.05 (*p* < 0.001) and the mean open time was 23.9 ± 7 ms and 17.7 ± 5 ms for the WT and p.Phe58Ser, respectively. The distribution of the events in Figure 5E summarized the mutation-dependent increase both in the open probability and in the channel amplitude.

## Discussion

In this article, we report a novel mutation (p.Phe58Ser) within the *KCNJ2* gene in an Italian proband of a family where both ASDs and cardiovascular disease segregate. The mutation was found in heterozygosis in the proband showing autism and a borderline SQT3 as well as in the father and paternal grandmother (both reported as not affected by ASDs). The grandmother family however add two ASDs cases plus a brother died by sudden cardiac arrest. Unfortunately, the DNA of these ASD relatives was not available, thus a segregation analysis of this mutation with this specific phenotype was not possible. The mutation causes the change of a highly conserved hydrophobic Phenylalanine (Phe) to a hydrophilic Serine (Ser) located in the N-terminal region of the protein. This is the second mutation in *KCNJ2* detected in a patient showing autism and SQT3 syndrome: the previously reported mutation (p.Lys346Thr) was detected in monozygotic twins and functional studies revealed an increase of the channel current amplitude due to an increase of its surface expression stability at the plasma-membrane, a reduction in protein degradation and altered protein compartmentalization (Ambrosini et al., 2014). The mutated channel reported here also showed an increase in the whole-cell current density as compared to the WT in the whole range of voltages tested, an hyperpolarization of the cell resting membrane potential and, indeed, a larger conductance revealed by single-channel recordings combined with a higher open channel probability.

The residue p.Phe58 resides within a short cluster of highly conserved basic amino acids (from amino acid 44–61) in the cytoplasmic N-terminus, which is required for Golgi exit (Hofherr et al., 2005; Houtman et al., 2014). It was demonstrated that the cell surface expression of Kir2.1 channel from the Golgi is controlled by a signal-dependent process that provides a mechanism to regulate the targeting of the channel to the sarcolemma and the transverse tubule at optimal densities for appropriate electrical signal transmission. The unusual Golgi exit signal is dictated by a tertiary structure localized within the confluence of the cytoplasmic NH2 and COOH terminal domains (Ma et al., 2011). The signal creates an interaction site that allows properly folded Kir2.1 channels to insert into clathrin-coated vesicles at the trans-Golgi for export to the cell surface (Xia et al., 2005). Despite the p.Phe58Ser mutation might be a modification factor for the above described tertiary structure owing to the fact that an apolar amino acid with a large aromatic residue is substituted by a polar one with a residue consisting in only a hydroxyl group, the mutated channel presented in this article does not appear to be retained in the Golgi apparatus (see Supplementary Figure S4).

As we did not detect an increase in the overall expression of the mutated channel in the total cellular lysate by western blotting analyses, the hypothesis of a different trafficking of the mutant channel was further investigated on cytosolic and membrane fractions of the transfected cells. The analyses revealed a trend of higher presence in the membrane of mutated channel as compared to the WT protein (see Supplementary Figure S2). This difference was not significant probably because of high variability and cell heterogeneity in transient transfection experiments. In order to overcome these limitations, a confocal imaging was performed and it confirmed the presence of the channel on the plasma-membrane. In order to gain direct and reliable information on Kir2.1 subcellular localization, we then used an anti-Kir2.1 antibody. Although immunofluorescence is an ideal method for qualitatively observing at protein distribution at individual cell level, effort was required to ensure that it was quantifiable. To this aim, we looked for a method to standardize the fluorescent signal emitted by the antibody bound to the Kir2.1 channel. Several trials using DilC12(3) were unsuccessful because this lipophilic fluorescent dye labels the cells membrane and yields a very good staining of *in vivo* cells. For *in vitro* acquisition, though, the staining was not homogeneous within the same coverslip, and for this reason we decided to rely on the fluorescent labeling of a constitutive membrane protein such as ZO-1 or to the WGA that binds to sugars in glycoproteins of the cells membrane. Setting these conditions, we were able to quantify and normalize the fluorescence associated to the protein of our interest. Our data showed that the fluorescence associated to the p.Phe58Ser bearing channel appeared to be slightly, but significantly higher, as compared to the WT, in both the conditions tested. This slight difference does not seem to be relevant for accounting as the main mechanism causing the gain of function. Rather, we are more prone to hypothesize that the increase in single channel conductance and in the open probability may be responsible for the effect observed in the whole cell measurements.

The clinical effect caused by the studied mutation is similar to those reported for other mutations in the Kir2.1 (e.g., p.D172N) detected in patients affected by SQT3S and/or atrial fibrillation without comorbidity with neuropsychiatric conditions (Xia et al., 2005). This is not unexpected considering the extreme genetic variability underlying the regulation of both myocytes and neurological (neurons and astrocytes) cells excitability. Due to the possibility of heterodimerization, the availability of different channels of the same family or the existence of reciprocal modulation between channels belonging to different families (e.g., Kir2.1 and Nav1.5; Milstein et al., 2012), it could be argued that some defects in one gene could be at least in part compensated by the presence of additional variations in one or more of other related genes. Thus, to unravel the real effect of each mutation on the final phenotype it could be necessary to exhaustively know the whole genetic background of patients. Whether Kir2.1 gain-of-function mutations might improve the susceptibility to ASDs remains to be clarified.

Although a functional effect of the Kir2.1-p.Phe58Ser mutation was demonstrated by our results, a direct role of the mutation in ASDs and/or cardiovascular disorders pathogenesis has still to be proved. This could be done only by the identification of new families with the mutation cosegregating with these diseases because, unfortunately, affected relatives of the proband were not compliant and did not participate to the study. Moreover, an incomplete penetrance could not be excluded owing to the detection of the variant in the father and grandmother that are not reported as affected by evident intellectual or social impairments (although it’s worthwhile to note that they never underwent to detailed psychological examination tests). Indeed, additional functional studies are needed to verify the results reported here in non-heterologous systems such as neurocytes, astrocytes or cardiomyocytes and the development of specific transgenic mouse models could give an indication on the phenotype specifically associated to the mutation. In fact, one has to consider that results related to protein function and expression are highly influenced by the cellular model selected and its environment, even more if the model used is an heterologous one, as it happens in the case of the majority of the studies related to the characterization of disease-linked mutation. That said, we emphasize the close relationship of the observed results with the cell model chosen and we do not exclude that other systems, perhaps more complex, may yield not exactly identical results.

In conclusion, the identification of an increased current density at a physiological range of voltages in the presence of mutations is consistent with the autistic phenotype, which is normally associated to an altered neuronal excitability: a Kir2.1 overexpression in the plasma-membrane has been previously reported to affect activity by lowering the resting membrane potential and therefore the cell excitability (De Marco García et al., 2011). This is the second variant detected in the *KCNJ2* gene in ASDs patients showing shortening of the QT interval and this recurrence suggests that individuals with such an altered channel density in the plasma membrane could be more prone to develop these diseases. Thus, the present article strengths the importance of a mutation screening of the whole *KCNJ2* gene in patients affected by ASDs.

## Data Availability

Datasets are available on request. With this statement, the authors intend that the raw data supporting the conclusion of this manuscript will be made available by the authors to any qualified researcher.

## Author Contributions

IR designed the patch clamp and immunofluorescence experiments, analyzed the data and participated in the drafting of the manuscript. AB performed the patch clamp experiments and analyzed the data. CV performed plasmids construction, amplification and sequencing as well as all cloning experiments. EC performed protein extraction and western-blot experiments. MB collected blood samples from participants and interviewed patients to reconstruct the family history. CMC wrote the clinical sections of the manuscript. RG designed immunoblotting and biochemical experiments, participated in the drafting of the manuscript. RC identified the mutation by sequencing, coordinated the study, wrote the manuscript. All authors contributed to the manuscript revision, read and approved the submitted version and are in agreement to be accountable for all the aspects of the work.

## Conflict of Interest Statement

The authors declare that the research was conducted in the absence of any commercial or financial relationships that could be construed as a potential conflict of interest.

## Abbreviations

SQT3: short QT Syndrome type 3
Kir: Inwardly-rectifying potassium channel.
