# Supplementary material for: A Novel KCNJ2 Mutation Identified in an Autistic Proband Affects the Single Channel Properties of Kir2.1
Source: Front Cell Neurosci. 2018 Mar 20;12:76. doi: 10.3389/fncel.2018.00076 (PMC5869910; doi:10.3389/fncel.2018.00076)
Supplement: Supplementary file 1 [file Data_Sheet_1.docx]

Supplementary Material

A novel *KCNJ2* mutation identified in an Autistic proband affects the single channel properties of Kir2.1

Anna Binda, Ilaria Rivolta^*^, Chiara Villa, Elisa Chisci, Massimiliano Beghi, Cesare Maria Cornaggia, Roberto Giovannoni, Romina Combi *

*** Correspondence:** Corresponding Author: [romina.combi@unimib.it](mailto:romina.combi@unimib.it)

ilaria.rivolta@unimib.it

# Immunoblotting analysis

The data we presented in the manuscript were obtained using pcDNA3.1-NT-GFP-TOPO vector (Invitrogen) for cell transfection procedure. This vector allowed to tag the *KCNJ2* gene with the GFP fused to the N terminus of the protein, obtaining pcDNA3.1-NT-GFP-TOPO-h*KCNJ2*-WT construct for the wild type channel or pcDNA3.1-NT-GFP-TOPO-h*KCNJ2*-p.Phe58Ser for the mutated form of the channel protein. In order to confirm the specificity of the western blot detection, we compared and merged the signals detected using the specific anti-Kir2.1 (mouse monoclonal [S21-32], 1:1000; Abcam [ab85492]) and the anti-GFP (rabbit polyclonal, 1:500, Life Technologies [A6455]) on the same membrane. Concerning this last blotting procedure, the nitrocellulose membrane was blocked with 5% milk solution prepared in PBS-Tween 0,1% and then incubated with anti-Kir2.1 antibody o/n at 4°C. The day after, the chemiluminescent signal corresponding to GFP-h*KCNJ2* fusion protein was detected by using a specific HRP-conjugate secondary antibody (anti-mouse IgG HRP-conjugate, 1:5000; GE Healthcare [NA931V]). Then, the blot was washed abundantly in PBS-Tween 0.1% and incubated with anti-GFP antibody 1h at RT. The GFP signal was detected by using a fluorochrome-conjugated secondary antibody (Affinity Purified Antibody Dylight™ Labeled Goat anti-rabbit IgG(H+L), 1:2000; KPL [072-05-18-06]).


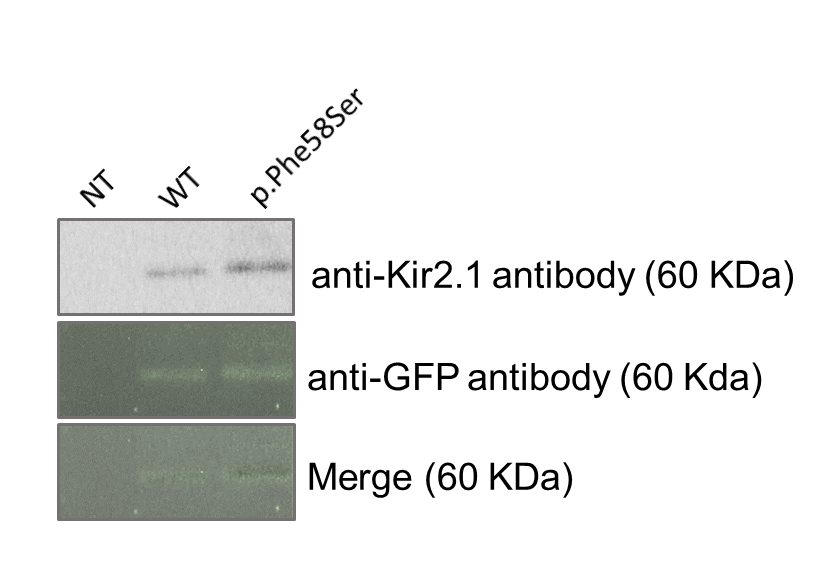


**Figure S1. Kir2.1 protein is fused to GFP in transfected cells.** Immunoblot analyses on total protein extracts from tsA201 cells untransfected (NT), transfected with pcDNA3.1-NT-GFP-TOPO-h*KCNJ2*-WT plasmid (WT) or pcDNA3.1-NT-GFP-TOPO-h*KCNJ2*-p.Phe58Ser plasmid (p.Phe58Ser). Anti-Kir2.1 and anti-GFP primary antibodies were used to hybridize the same membrane and the corresponding chemiluminescent (Kir2.1) and fluorescent (GFP) signals were detected with properly conjugated secondary antibodies. The merged image (merge) showed the co-localization of Kir2.1 and GFP bands (60kDa).

For cytosolic and membrane fractions separation analysis, total protein extracts were obtained by lysing 5x10^6^ cells with 1X RIPA lysis buffer (50mM Tris-HCl pH 7.4, 150mM NaCl, 1% Triton X-100, 0.1 % SDS) added with 1mM DTT, 1mM EDTA and EGTA, and 1.5% Protease Inhibitor Cocktail and Phosphatase Inhibitor Cocktail, while cytosolic and membrane protein fractions were obtained by using the Mem-PER™ Plus Kit (Thermo Scientific), following manufacturer’s instructions. Protein extracts were quantified by Bradford assay (Sigma Aldrich). 10 µg of total, cytosolic or membrane protein extracts were loaded on NuPAGE Bis-Tris pre-casted mini gels (Life Technologies), following manufacturer instructions. Blotting onto nitrocellulose membrane (Life Technologies) was performed using iBlot System 2 (Life Technologies). Nitrocellulose membranes were blocked with 5% milk solution prepared in PBS-Tween 0,1%, and then incubated with anti-E-Cadherin (mouse polyclonal, 1:1000, BD Biosciences [610182]) antibody overnight at 4°C, and with anti-GFP (rabbit polyclonal, 1:500, Life Technologies [A6455]), and anti-β-actin (mouse monoclonal [AC-15], 1:5000, Sigma Aldrich) antibodies for 1 hour at room temperature. Membranes were washed 3 times in PBS-Tween 0,1% and then incubated with the following secondary antibodies: ECL anti-mouse IgG HRP linked (1:5000, GE Healthcare) or ECL anti-rabbit IgG HRP linked (GE Healthcare, 1:5000). After 3 washes in PBS-Tween 0,1%, Liteblot® Extend Long Lasting Chemiluminescent Substrate (Euroclone) was added to the membranes and chemiluminescent signal was digitally acquired by GBox (Syngene).


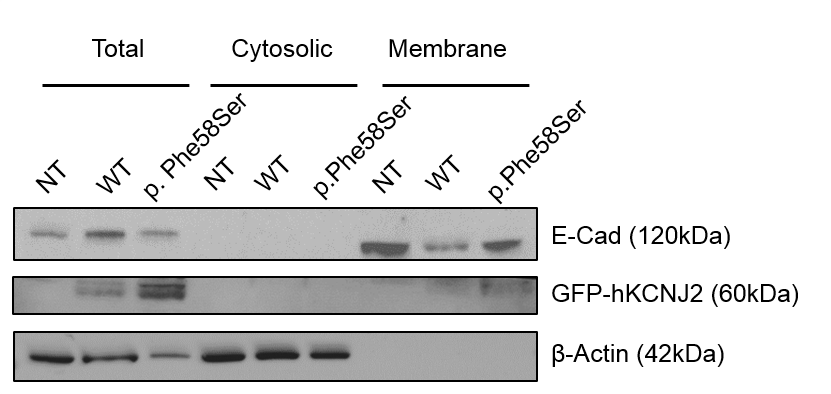


**Figure S2. Quantification of GFP/KCNJ2 protein in cytosolic and membrane fractions of transfected cells.** Immunoblot analysis on total, cytosolic and membrane protein fractions from tsA201 cells transfected with pcDNA3.1-NT-GFP-TOPO-h*KCNJ2*-WT plasmid (WT) or pcDNA3.1-NT-GFP-TOPO-h*KCNJ2*-p.Phe58Ser plasmid (p.Phe58Ser) and untransfected tsA201 cells (NT) is showed. E-Cadherin (E-Cad) and β-Actin were used as endogenous controls of membrane and cytosolic protein extracts, respectively.

**2. Electrophysiology**

The data we presented in the manuscript were obtained using pcDNA3.1-NT-GFP-TOPO vector (Invitrogen) for cell transfection procedure. This vector allowed to tag the *KCNJ2* gene with the GFP fused to the N terminus of the protein, obtaining pcDNA3.1-NT-GFP-TOPO-h*KCNJ2*-WT construct for the wild type channel or pcDNA3.1-NT-GFP-TOPO-h*KCNJ2*-p.Phe58Ser for the mutated form of the channel protein. These constructs gave us the opportunity to correlate unequivocally the fluorescence detected with the presence of the ion channel of interest.

In order to make sure that the fusion of the GFP to the N-term of the channel did not alter the electrophysiological properties of the channel itself, we performed parallel whole-cell experiments in which we recorded families of current traces from tsA201 cells transfected with 1 µg either of pcDNA3.1-NT-GFP-TOPO-h*KCNJ2*-WT (in the figure as GFP-*KCNJ2*-WT) or the original pCMS-EGFP-h*KNCJ2*-WT (in the figure as *KCNJ2*-WT, kindly provided by Prof. Minoru Horie, Shiga University of Medical Science, Japan). Mind that in this second case the GFP is present in the vector, but not fused to the ion channel. For the mutant channel the vectors tested were pcDNA3.1-NT-GFP-TOPO-h*KCNJ2*-p.Phe58Ser (in the figure as GFP-*KCNJ2*-p.Phe58Ser) and pCMS-EGFP-h*KNCJ2*-p.Phe58Ser (in the figure as *KCNJ2*-p.Phe58Ser). Even though the EGFP tag of p.Phe58Ser channels seems to lead to increased mean current density, this effect is not statistically significant (p=0.862), as well as the apparent decrease in the current density observed in the comparison between the EGFP tag and no tag WT Kir2.1 (p=0.123).

Solution used and protocols applied were already described in the Materials and Methods section of the manuscript.

As showed in the Figure S3 by the overlap of the curves of the two pairs of constructs, the fusion of the GFP to the N terminus of the channel did not alter the properties neither of the WT, nor of proteins harboring the mutation.


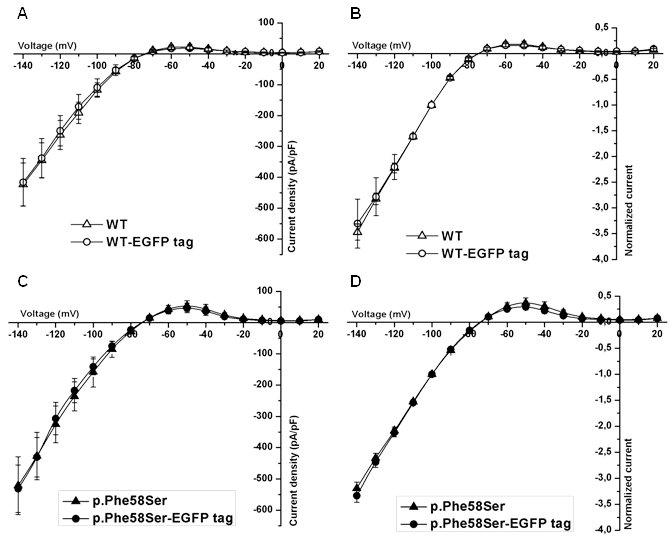


**Figure S3.** **Comparison of the current/voltage relation (I/V) of the *KCNJ2* protein fused or not fused with the GFP tag**. Panel A-B: I/V expressing the current density or normalized current (to -100 mV) of the *KCNJ2*-WT vs the GFP-*KCNJ2*-WT. The complete overlap of curves suggested that the channels behaved similarly. Panel C-D: I/V expressing the current density or normalized current (to -100 mV) of the *KCNJ2*- p.Phe58Ser vs the GFP-*KCNJ2*- p.Phe58Ser. Also in this case the overlap is observed. Data are presented as mean±SEM, at least 20 cells for condition were recorded from 5 different experiments

**3. Immunofluorescence**

As the residue p.Phe58 resides within a cluster of highly conserved basic amino acids (from amino acid 44 to 61) required for Golgi exit, we investigated whether its substitution with a Ser may affect the retention of the mutated ion channel in the Golgi apparatus. To this aim we incubated the transfected tsA201 cells overnight at 4°C with Anti-Kir2.1 (mouse monoclonal, [S21-32], 1:100, Abcam [ab85492]) and anti-GM130 (rabbit polyclonal, 1:200; Proteintech) in GDB. The secondary antibodies were conjugated with Alexa fluorophores (goat anti-mouse Alexa Fluor 488, goat anti-rabbit Alexa Fluor 568; Invitrogen) diluted in GDB, at room temperature for 1 hour. 1 µM of 4',6-diamidino-2-phenylindole (DAPI) in PBS stained cell nuclei (5 min). GM130 is peripheral cytoplasmic protein part of the cis-Golgi matrix, thus it is considered a good marker for the Golgi apparatus. Confocal laser scanning microscopy was used to study the presence of the Kir2.1 WT or mutated channel within the Golgi cisternae. Images were acquired by an LSM710 inverted confocal microscope equipped with a Plan-Neofluar 63×1.4 oil objective (Carl Zeiss, Germany) at λem=610 nm to detect GM130 on the plasma membrane, λem=488 nm to detect the ion channel, and λem=460 nm to detect the nuclei.


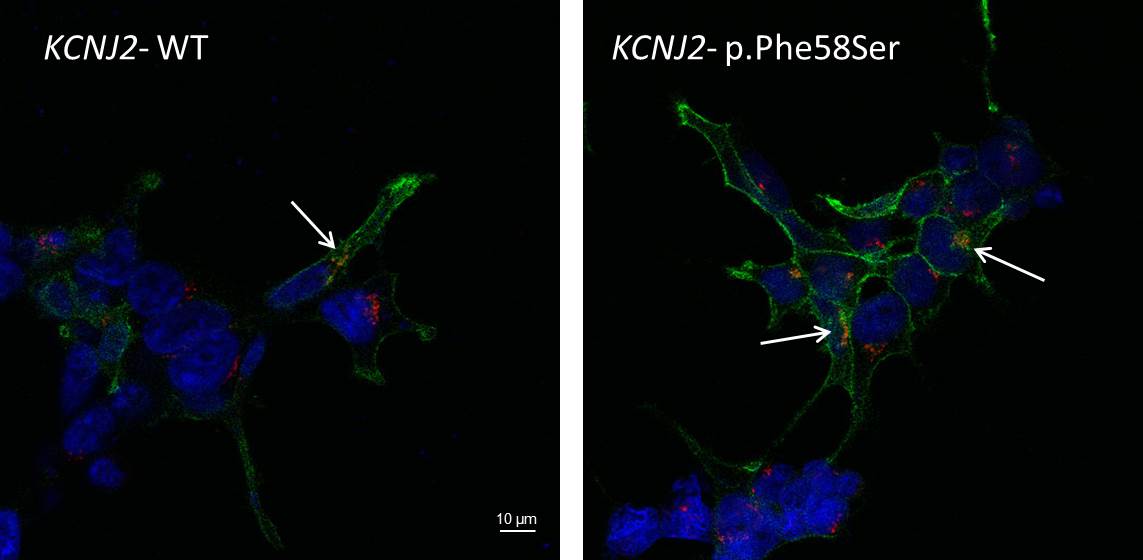


**Figure S4. The *KCNJ2*- p.Phe58Ser mutation does not affect the ion channel transit within the Golgi apparatus.** Confocal images of tsa201 cells revealing a comparable level of co-localization (white arrows) between the Kir2.1 channel (in green) and the GM130 protein (in red) regardless the presence of the mutation.
